# Breast-i Is an Effective and Reliable Adjunct Screening Tool for Detecting Early Tumour Related Angiogenesis of Breast Cancers in Low Resource Sub-Saharan Countries

**DOI:** 10.1155/2018/2539056

**Published:** 2018-04-04

**Authors:** Frank Naku Ghartey, David Watmough, Samuel Debrah, Martin Morna, Akwasi Anyanful

**Affiliations:** ^1^Department of Chemical Pathology, School of Medical Sciences, University of Cape Coast, Cape Coast, Ghana; ^2^Highland Innovation Centre, Inverness, UK; ^3^Department of Surgery, School of Medical Sciences, University of Cape Coast, Cape Coast, Ghana; ^4^Department of Medical Biochemistry, School of Medical Sciences, University of Cape Coast, Cape Coast, Ghana

## Abstract

**Background:**

What cheaper alternative breast screening procedures are available to younger women in addition to clinical breast examination (CBE) in Sub-Saharan countries? In 2009, we first described BreastLight for screening and reported high sensitivity at detecting breast cancer. Due to limitations of BreastLight, we have since 2014 been using the more technologically advanced Breast-i to screen 2204 women to find cheaper screening alternatives.

**Methodology:**

First, the participant lies down for CBE and then, in a darkened room, Breast-i was placed underneath each breast and trained personnel confirm vein pattern and look out for dark spot(s) to ascertain the presence of suspicious angiogenic lesion(s).

**Results:**

CBE detected 153 palpable breast masses and Breast-i, which detects angiogenesis, confirmed 136. However, Breast-i detected 22 more cases of which 7 had angiogenesis but were not palpable and 15 were missed by CBE due to large breast size. Overall confirmed cases were 26, with Breast-i detecting 7 cases missed by CBE. Breast-i and CBE gave sensitivities of 92.3% and 73%, respectively.

**Conclusion:**

Breast-i with its high sensitivity to angiogenesis, reliability, and affordability will be an effective adjunct detection device that can be used effectively to increase early detection in younger women, thereby increasing treatment success.

## 1. Introduction

Breast cancer, a genetically and clinically heterogeneous disease [1], is the most frequently diagnosed cancer worldwide and a leading cause of cancer death among women [2]. In 2012, nearly 1.67 million new cases of breast cancer were diagnosed worldwide representing about 12% of all new cancer cases and 25% of all cancers in women [3]. Of this, about 883,000 were reported in middle to low income regions and 788,000 cases in developed regions with deaths of about 324,000 and 198,000, respectively [4]. Breast cancer incidence has been increasing yearly worldwide and the Sub-Saharan region of Africa is no exception as indicated by reports from American Cancer Society [5], Cote d'Ivorie, [6], Uganda [7, 8], Nigeria [9], and Ghana [10].

In Ghana, breast cancer is now the most common malignant disease in women accounting for a majority of cancer related deaths [11]. Reports of breast cancer burden in the Sub-region at treatment centres include late presentation, advanced metastatic breast cancer, poor survival rate [12] and higher prevalence of triple negative breast cancer [13–16], Furthermore, other reports indicate more cases occurring in premenopausal women [17, 18] with peak incidences approximately 10 years earlier than the Western counterparts [18, 19]. This therefore calls for increasing awareness and screening for early detection to improve therapeutic success. Mammography, the gold standard, is not easily applicable in Ghana and most Sub-Saharan countries due to its unavailability, unreliable power, lack of manpower, and more importantly nonusage in women below 40 years old with dense breast tissue. Thus Clinical Breast Examination (CBE) to detect palpable lumps has been recommended as the first procedure for assessing breast cancer in low resource Sub-Saharan countries including Ghana [20]. However CBE sensitivity is low and ranges from 44.6% to 65.9% [21], perhaps due to its dependence on the presence of palpable masses and experience of the clinician. CBE is also unable to distinguish between benign and malignant tumours in early stage disease and is therefore recommended to be used alongside ultrasonography, mammography, and histopathology whenever applicable during periodic examinations for improved sensitivity [22–24].

The mean age for breast cancer diagnosis in Ghana is 39 years [18] suggesting that screening should start ten years earlier. However, since CBE has low sensitivity and mammography is also not recommended for women under 40 years, it is therefore imperative that we find alternative cheaper, reliable, and more effective ways to screen women in Ghana.

**Figure 1:**
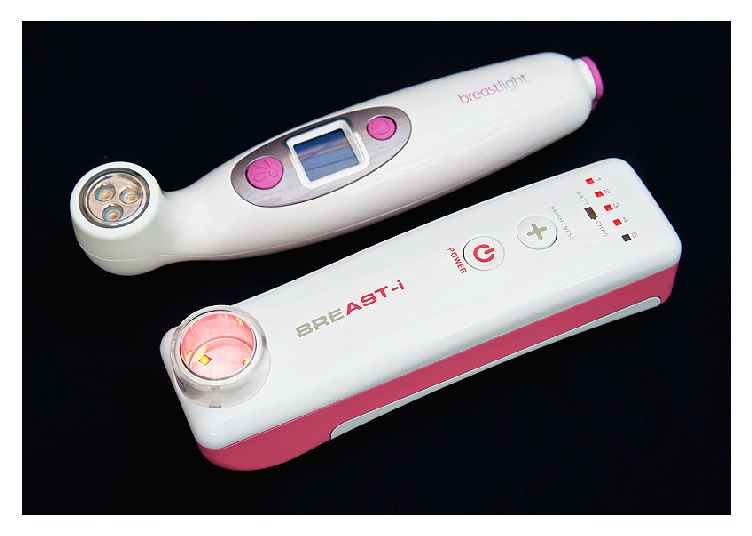
*Breast-i and BreastLight*. Breast-i when fully charged can be used to examine the breasts of about 70 women making it useful and accessible in areas with unreliable electricity. Usage requires just a dark room and a well-trained handler; hence it is inexpensive to use. Breast-i is equipped with five adjustable light intensities that enable usage on various skin tones, breast sizes and densities, and pregnant and lactating women. The advance sensor technology also protects the eyes of users.

The use of transillumination as an aid in the diagnosis of breast lesions was first shown by Cutler in 1929 [25] followed by Angquist and colleagues in 1981 [26]. Another study by Watmough in 1982 [27] showed that due to the associated angiogenesis that supplies oxygen and nutrients to cancer cells, optical images of breast cancer can be seen when red blood cells (oxyhaemoglobin) absorb light at about 615 nm. This lead to the production of an affordable compact optical device called the BreastLight to aid in the early detection of breast cancer. Breastlight and the more technologically advanced fourth-generation Breast-i (Figure 1) produced upon recommendations from BreastLight results are handheld optical devices developed as an “internal sight” to increase breast awareness and help women better spot changes in their breasts. Breast-i which we used in this study, when placed under the breast in a dark room, operates by emitting high intense red light within the region of 614–620 nm, which illuminates the breast. However, due to absorption of the light by haemoglobin, the patterns of blood vessels are seen as dark lines. The degree of light absorption determined by the number of blood cells per unit volume of breast tissue produced shadows in the case of blood filled cysts, abscess, haematomas, and neoplastic tumours. Thus a normal healthy breast will appear red with uniform brightness accompanied by a well-defined black vein structure. A benign lesion which also has no associated angiogenesis will not give the dark shadow. However, any angiogenesis or similar breast abnormality will give rise to a dark area or shadow enabling the detection of suspicious lesions even if it is nonpalpable. A test at Sunderland hospital showed that Breastlight detected 12 out of 18 malignant tumours (67%) and gave a specificity of 85% (240/282 breasts) [28]. A smaller study involving 310 females shows almost equal sensitivity between Breastlight and mammography in detecting breast cancer [29]. Another report using 500 women from Iran also reported the efficacy of BreastLight in detection of breast changes [30]. However, BreastLight could not penetrate easily dense breasts of young African women and women who were very dark in complexion. It was also not very effective in detecting small lesions in women with large sized breasts and in most women who were either breastfeeding or in the third trimester. Furthermore, prolonged exposure with BreastLight also generated heat which made the women uncomfortable. This led to the generation of Breast-i to meet these challenges and in this study we report our results with Breast-i after examining over 2000 women. Our main objective is to evaluate the efficacy of Breast-i as an “internal sight” and adjunct to CBE in especially the young female population to enhance early detection. Thus the present study will attempt to answer the following questions. (i) Will Breast-i be an effective screening and diagnostic tool in the detection of clinically suspicious lesions in the young black population? (ii) Can Breast-i detect nonpalpable masses as well as small masses in large sized breast which may be missed by CBE? (iii) Can Breast-i predict a suspicious mass as benign or malignant? (iv) What will be the sensitivity and specificity of Breast-i in comparison with CBE? (v) Will our result recommend the use of Breast-i as an alternative/complementary mass screening tool (not replacing) especially in the younger generation in low resource Sub-Saharan countries including Ghana? Giving the findings with BreastLight, we believe that Breast-i may provide results that will inform policy makers to take a second look at breast screening and breast cancer management policies.

## 2. Methodology

### 2.1. Study Area and Design

This is an experimental study carried out from September 2014 to February 2017. Participants were recruited from Central and Greater Accra regions of Ghana but mainly from Central because of location and accessibility to a particular age group in the population. Secondly, we wanted participants found to have suspicious lumps to be able to have access to treatment centres for further evaluation and possible treatment if needed. Lastly, due to limited funding, we could not include more participants as we desired.

### 2.2. Ethical Considerations

The study was a partnership between Mammocare, a nongovernmental Organization in Ghana, and Highland Innovations of Scotland, producers of the Breast-i on one side, together with Ministries of Women and Children and Health of Ghana. The Ministries' Joint Trustee Board for breast cancer awareness and early detection having been approved by the Ministries' Ethical Review Committee granted authority to Mammocare to conduct the study. Mammocare was required to organize an awareness talk, explain all procedures involving the Breast-i, and respond to participant's questions before the screening process. Discussions included painless and harmless procedural process, no coercion, ability to withdraw at one's own will, no invasion of privacy or involvement of deception, and absence of monetary rewards. After all of this, only willing participants who picked and completed a consent form were included in the study. The Breast-i had been approved by the Ghana Standards Authority as a breast screening device.

### 2.3. Selection of Representative Participants and Data Collection

In Central Region, we contacted the Women's Group of Churches for the awareness talk and participation. We also contacted the Women's Commissioner and Female Hall Presidents in the tertiary institutions. Lastly, in October, which is the breast cancer awareness month, we set up temporary stations all over town where women walk in for free screening. In the Greater Accra region, our activities are restricted more to the churches upon invitation. Willing females between 18 and 70 years who consented after the talk or after thoroughly explaining the procedures of CBE and Breast-i first underwent CBE followed by examination with the Breast-i in an adjoining room. In addition to screening, participants respond to questionnaires which captured health status, knowledge and perception of breast cancer and its symptoms, and socioeconomic and geographical information. All in all, we had accurate information on 2204 ladies who were screened with CBE and Breast-i over the period.

### 2.4. CBE and Breast-i Examination

**Figure 2:**
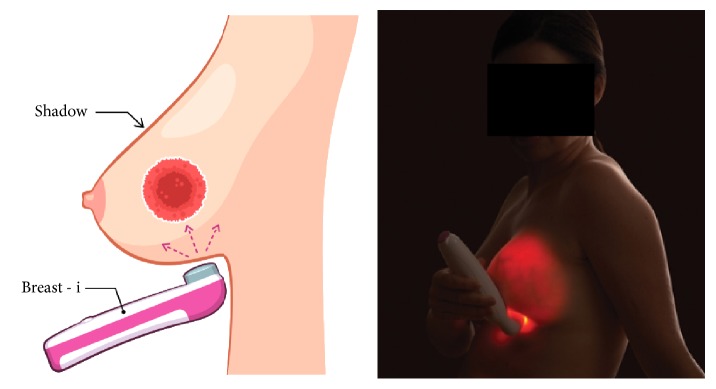
*Illustration of how to use the optical device*. In a darkened room, switch Breast-i on and place it underneath the breast. Adjust light intensity as required and view each breast looking out for any spots or shadows. Sometimes it is necessary to place the Breast-i on top of the breast and view underneath the breast directly or use a mirror. Light is scattered through the breast tissues giving it a uniform pink or red colour with blood vessels seen as dark lines.

CBE was first used to examine the breast and any suggestion of the presence of a palpable lump or suspicious lesion(s) was noted as per the information on the questionnaire. Next, in a darkened room Breast-i was pressed gently against the inferior surface of the female breast for illumination (Figure 2) and identification of angiogenesis. Any dark spot(s) or shadow(s) within or on the superior surface of the breast, if any, indicates the possible presence of a suspicious lesion that may need further evaluation. The significance of a dark spot/shadow arises because the transmitted light is strongly absorbed by blood vessels and angiogenesis surrounding possible cancers which then gives rise to a spot/shadow seen on the superior breast surface. It has to be appreciated that though the device was developed mainly to detect cancers which have associated angiogenesis, blood filled cysts, abscesses, and bruises can all give rise to dark shadows. Patients with findings in any of these categories were referred to our surgical team for confirmatory diagnosis and treatment.

### 2.5. Confirmation of Shadows and Palpable Masses

All cases of either dark spots/shadows and/or palpable masses were considered as suspicious lesions and referred to the collaborating surgeons at the regional health facilities for histopathological confirmation. Results of the confirmation enabled the assessment of the efficacy of Breast-i as an adjunct screening tool which could be adopted by low resource Sub-Saharan countries in addition to CBE especially for the younger population. Follow-ups by phone were made to participants who were positively diagnosed with breast cancer to encourage them to attend the health centres and comply with the treatment regimen prescribed. This was the best we could do due to the limited resources at our disposal.

## 3. Results

### 3.1. Demographics

The mean ages of the study participants were 34 and 41 years for Central and Greater Accra regions, respectively. This is because the study was skewed towards screening younger women as we wanted to determine the efficacy of Breast-i in this group who could not use mammography services. In central region, most of the participants were either students in tertiary institutions or staff of secondary and tertiary institutions. Hence, most of the participants were educated, lived in the urban areas, and had above adequate knowledge of breast cancer. In Greater Accra region, most of the participants were from churches and the figures are quite evenly spread. All of this is summarized in Table 1.

### 3.2. Breast-i Is Capable of Detecting Blood Vessels in the Breast

**Figure 3:**
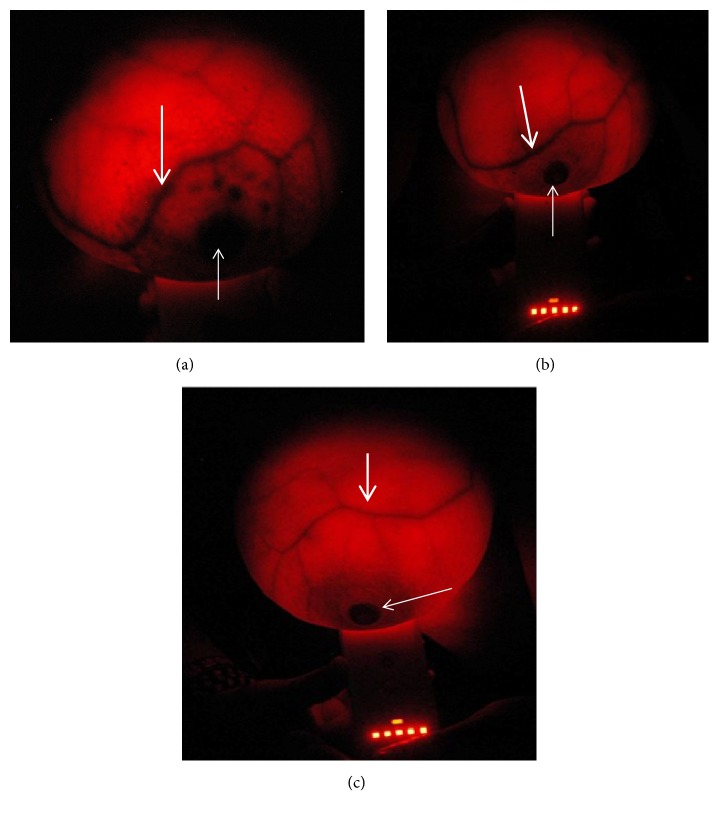
*Breast-i images of the breasts of three participants*. The thick arrows show the vein pattern of the right breast of a 46-year-old participant (a), 30-year-old participant (b), and 20-year-old participant (c). The thin arrows show the circular area around the nipples which is normally dark due to normal pigmentation of the areola. Breasts in both the 30 and 20 year olds were dense and required maximum intensity light for visualization. These breasts are normal and devoid of any suspicious shadows/spots.

Figures 3(a), 3(b), and 3(c) show the illumination of the left breasts of three participants aged 46 (a) and 30 (b) and 20 (c) with Figure 3(c) being the most dense. The denser the breast the greater the intensity of light required ((b) and (c)). Light from Breast-i which is emitted at a wavelength between 614 and 620 nm is strongly absorbed by the haem pigment present in blood. Therefore, while the breast appears red with uniform brightness, the blood vessels will appear black showing a clear vein pattern (thick arrows). The nipples are shown with thin arrows and the circular area around the nipple (areolar) in most breasts observed was dark due to normal pigmentation of the areola. Light from BreastLight is unable to penetrate well the dense breasts of younger ladies who were also dark in complexion. However, breast-i penetrates quite well as seen in the image of a 20 year old in Figure 3(c). Breast-i, unlike BreastLight, can also be used for females who were in the third trimester and those who were actively breastfeeding, both of whom have milk in the breasts. It was also observed that the vein pattern is different for each breast and it was common for one pattern to be more pronounced than the other. These images therefore show clearly that Breast-i can be used as a blood vessel detecting device which could become very handy when it comes to detecting angiogenesis around tumour cells.

### 3.3. BreastLight/Breast-i Can Detect Angiogenesis of Neoplastic Lesions in the Breast

**Figure 4:**
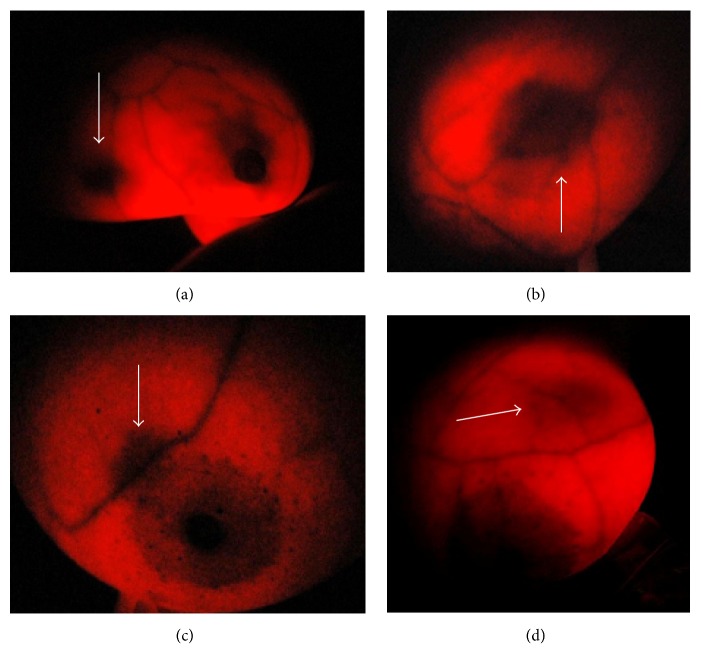
*Images of lesions detected by Breast-i*. (a) Impalpable lesion (arrowed) in a 36 year old near the axillary tail of breast. Tissue analysis revealed a malignant lesion. (b) Palpable recurring bloody cyst (arrow) in a 47 year old. Tissue analysis revealed a malignant lesion. (c) Leaky blood vessel (arrow) in a 50 year old. No malignancy associated. (d) Thick white arrow points at impalpable tender lesion in a 28 year old. The tissue analysis was consistent with mastitis.

Neoplastic lesions have the ability to form new blood vessels (angiogenesis) from existing ones to support the characteristic rapid cell divisions and growth. Angiogenesis also facilitates the spread of malignant tumour cells to distant organs (metastasis). If Breast-i has the capability of detecting haem and blood vessels then it should be capable of showing angiogenesis around neoplastic lesions.  Figure 4(a) is a Breast-i image of a 36 year old with an impalpable lesion. Breast-i detected a dark spot/enhanced vascularization (arrow) which was later confirmed as malignant (Table 3).  Figure 4(b) was palpable lesion in a 47 year old, which was also detected with the Breast-i (arrow) and was confirmed malignant (Table 3). The darkened area in Figure 4(c) in a 50 year old was not palpable and was neither confirmed to be malignant nor benign. Since it resolved within a month, we believe that it may have been a leaking blood vessel.  Figure 4(d) shows a marked diffuse shadow at the one o'clock position in a 28 year old which turned out to be an infection (mastitis). The advantage of the Breast-i is our ability to take photographs and compare them to follow-up screening shots to ascertain whether the shadows are increasing, decreasing, or becoming more prominent. Lastly, in a blind test at the Cape Coast Teaching Hospital in Ghana using 15 patients, Breast-i correctly identified the eight known cancer cases and dismissed the other seven. These results show that Breast-i can be reliably used as an adjunct screening tool to detect breast cancer and other breast changes or abnormalities.

### 3.4. Breast-i Predicts and Detects Source of Bloody Nipple Discharge

**Figure 5:**
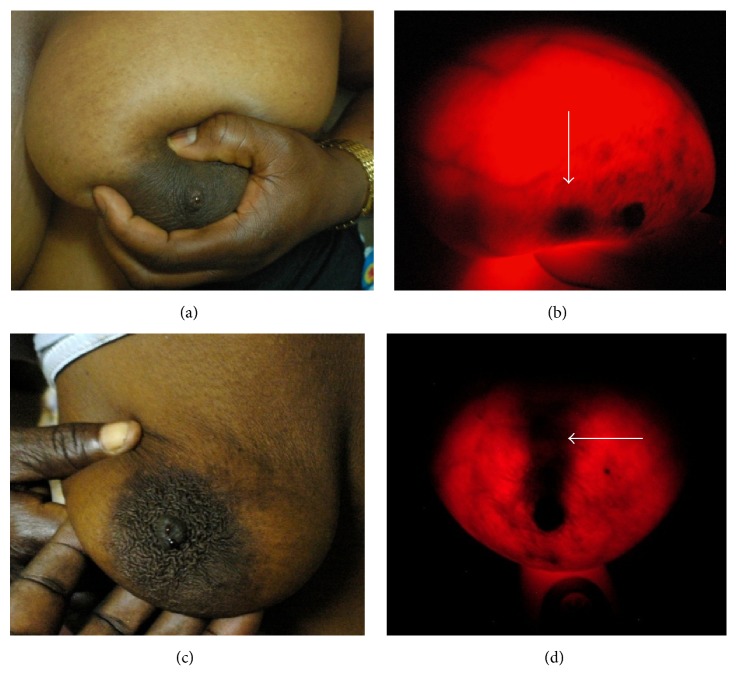
*Breast-i locates possible sources of bloody nipple discharge*. (a) and (c) are photographed images of bloody nipple discharge when breasts were expressed. Expression was requested upon detection of suspicious lesions from Breast-i examination. (b) and (d) white arrows show possible lesions that may be responsible for the bloody discharge.

Bloody nipple discharge is one of the symptoms of possible breast cancer that should always be evaluated further. In some cases the bloody discharge may not be accompanied by a palpable mass. We therefore decided to ascertain whether Breast-i could predict or detect the source of the bloody discharge. The images in Figures 5(b) and 5(d) clearly show darkened spots in breasts indicative possible positions of suspicious lesions (arrows), even though they were not palpable. Participants were requested to express the breasts and Figures 5(a) and 5(c) correspondingly show bloody discharge from the nipples (Table 3). The discharge was easily expressed in patient (c) compared to patient (a). In the case of (d) the darkened area was large and extended from the nipple upwards so is it possible that Breast-i may have some quantitative properties at least with bloody discharges. Furthermore, using Breast-i as a guiding tool in the case of (d), the surgeon withdrew blood from the suspected angiogenic area and one could see the shrinkage and fading of the dark shadow a few minutes later after the blood withdrawal. These results further confirm the usage of Breast-i as a reliable adjunct screening device capable of picking nonpalpable lesions with associated angiogenesis.

### 3.5. Breast-i Can Serve as an Adjunct to Clinical Breast Examination (CBE)

The average age of breast cancer diagnosis in Ghana is 39 years [18] meaning that screening should start in the early 30s. Due to unavailability and unsuitability of mammography for this age group, screening is mainly done by CBE, which is dependent on the presence of palpable masses and the experience of the examiner. Since we are proposing Breast-i as an adjunct screening tool to complement the weaknesses of CBE, it is imperative that we compare its efficacy to CBE.  Table 2 shows that of the 2204 participants that were examined by both Breast-i and CBE, CBE palpated 153 lumps, out of which 136 were also detected by Breast-i. However, Breast-i was able to detect additional 22 suspicious lesions which could not detected by CBE. Thus, overall, Breast-i was able to detect 5 more suspicious cases than CBE (158 versus 153), making it in our opinion a very reliable detection tool.

### 3.6. Breast-i Can Detect Cancer Cases Missed by CBE

Of the 22 suspicious cases detected by Breast-i alone (Table 2), 7 were nonpalpable but angiogenic and out of these 4 were confirmed as cancer cases (Table 3). The remaining 15 we believe should have been palpable but were missed because of the size of the breast lesion relative to the large size of the breast. However, they were picked up with Breast-i and 3 out of these 15 turned out to be malignant. Thus a total of 7 malignant cases picked by Breast-i were missed by CBE. The minimal size lesion that Breast-i has ever detected had a diameter of about 9 mm, which is not easily palpable, and an experienced handler should be able to palpate lesions greater than or equal to 12 mm.

Overall, 26 breast cancer cases were confirmed at the referral and treatment centre and Breast-i detected 24 giving it a sensitivity of 92.3% at detecting breast cancer. CBE picked 19 cases and thus had a sensitivity of 73% (Table 3).  Table 4 shows the distribution of the cancer cases in the various age groups. Almost 35% of the cases were below 45 years old, which follows the current trend seen in Black population in Sub-Saharan Africa. These results show that Breast-i is far more superior and more reliable at detecting malignant tumours in the breast than CBE and we recommend its usage as an adjunct screening device.

## 4. Discussion

Breast cancer is a devastating disease that impacts in a monstrous destructive way victims, families, and developing nations as a whole. Therefore any procedure or device that will increase early detection and therefore improve survival outcome, even if it is for one person, should not be ignored but evaluated and adopted if tests confirm its efficacy. In Sub-Saharan Africa, breast cancer, the most diagnosed cancer in women, most likely occurs in premenopausal women, with peak incidences around 39 years [17–19, 31, 32] warranting screening from early 30s. Mammography, the gold standard, aside from being not readily available and lacking skilled manpower, is not recommended for ladies below 40 years. With CBE being unreliable as sensitivities vary [21], there was the need to find another effective mode of screening for breast lesions, taking into consideration affordability, availability, and acceptability especially in low income countries, which have other health problems too numerous to list here. This leads to the development of a light based method that can see “the inside” of the breast to complement the weaknesses of CBE. Thus at the 2nd Annual Africa Breast Cancer Conference, Cairo, Egypt, in 2009 we described for the first time the combined use of BreastLight and CBE and reported a combined sensitivity of 96% for detecting breast cancer. However with problems of heat, low transmission in dense and dark breasts, and inability to use in pregnant and lactating mothers amongst others, Breast-i, a more technologically advanced form of BreastLight, was developed. We must state here emphatically that both Breast-i and BreastLight are not diagnostic devices and are to be used as an adjunct, but not a replacement to current screening procedures.

Participants for this study were mainly drawn from tertiary institutions in Cape Coast and from churches in Accra, the capital city of Ghana. Most of the participants in Cape Coast were having or had had tertiary education as they were affiliated to universities and had adequate knowledge of breast cancer (Table 1). Surprisingly, with all the education and knowledge, majority of them were screening for the first time, a trend which has also been reported in other Sub-Saharan countries for educated ladies including even hospital workers [33, 34]. In Accra, the educational level and knowledge of breast cancer represent what is generally seen across the country [35] as no particular group was targeted. Since the focus was to determine the efficacy of Breast-i in younger women with denser breasts and who are most likely pregnant or lactating, about 50% of the participants were below 35 years old and 70% below 45 years old (Table 4). Within this age group 10 cancer cases representing 38.4% of the cancer cases were confirmed (Table 4). This corresponds to a penetrance of 0.45% which is quite similar to 0.53% reported in an earlier study [18]. However, for this age group, both penetrance results are worrisome as they reflect an increasing incidence of breast cancer in the country.

Breast-i was developed to overcome the deficiencies of BreastLight. It must therefore produce the expected images for every lady screened irrespective of age, skin tone, breast density and size, lactation, or late pregnancy. Figures 3(a)–3(c) show clearly the vein patterns of participants aged 46, 30, and 20 years, respectively. These breasts shown in the images were of average size and tone. Breast-i, in addition, comfortably coped with extra-large breasts and breasts of very dark skinned ladies. It was also able to produce clear images for ladies as young as 14 years old who were brought by their parents for screening (not shown for ethical reasons). Furthermore, during screening, ladies are asked to look at the breasts and be on the lookout for any dark spots. Aside from being fascinated by the vein pattern, the fact that they played a role in their own health check gives them the needed assurance and confidence in the results. Several times, requests were made for repeated exposure for assurance of the absence of a perceived spot. The usage of Breast-i in the dark setting was also welcome, as ladies who feel squeamish exposing their breasts in broad daylight especially to male personnel for manual palpation are more comfortable in this setting. An addition advantage of having Breast-i as confirmed by hospital personnel is its usage in localizing difficult-to-see blood vessels for blood withdrawal or setting IVs in both adults and children. Though nobody has reported it yet, we believe that Breast-i can be used to examine the scrotum and testis and the blood vessels around them.

Breast-i was developed as an adjunct tool to see the “inside” of breasts for angiogenic detection of suspicious lumps. As seen from Figure 4, Breast-i proved to be a reliable device by detecting breast masses, blood leakages, abscess, and blood filled cysts. When used accurately, Breast-i rarely misses lesions greater than 14 mm even within large sized breasts and an experienced handler can detect lesions approximately 12 mm in size. This enabled the detection of angiogenic lesions in breasts that were not palpable especially when the breasts are of a larger size (Table 3). This is an advantage of Breast-i over CBE and, in this case, 3 out of the 15 suspicious lesions in such large breasts turned out to be cancerous (Table 3). By requesting expression of these breasts, participants sometimes saw for the first time a bloody discharge coming out of their nipples (Figure 5). Furthermore due to the possibility of taking photographs of the spots, Breast-i can be used to follow the progression of the disease. In a few cases where the spots were barely visible or blurred, participants were asked to report a month later and photographs taken at both visits were compared in order to form an opinion. Likewise Breast-i can also be used to follow the progression of treatment by comparing pretreatment and posttreatment photographs to ascertain if the spots are shrinking and/or fading.

Table 2 shows that Breast-i was able to pick 136 suspicious cases compared to the 153 picked by CBE. This is not surprising since Breast-i is designed to pick angiogenesis and will have a higher miss rate for fatty lumps and nonbloody lumps. This is not a disadvantage in real practical terms since nonangiogenic lumps often turn out to be benign, have no grave clinical consequences, and should not be too much of an immediate concern. By the same principle, when a lesion is picked up by Breast-i, then it warrants further evaluation as it is potentially cancerous. This is confirmed in Table 2, where of the 22 suspicious cases missed by CBE but picked by Breast-i, 7 were found to be cancerous (Table 3). Overall Breast-i detected 24 out of the confirmed 26 cancer cases giving it a sensitivity of 92.3%, whilst CBE detected 19 out of 26 for a sensitivity of 73%. The sensitivity of Breast-i is comparable to the 93% reported by Labib et al. [29] using BreastLight on 310 women; however in his case 81% of the participants were referred accounting for the high sensitivity.

Table 4 links the number of participants, age grouping, and breast cancer cases together. The 26 confirmed cases out of a population of 2204 give about a 1.1% penetrance. In a previous study of 3000 participants in 5 regions of Ghana, we reported a penetrance of 0.76% [18]. Our methodology was CBE and, from Table 3, it is quite possible that we may have missed some positive cases. It is also possible that our higher penetrance may be due to our numerous breast cancer awareness campaigns that may be bringing out more women for screening. The figure however is not far-fetched because a study in 2008 puts the penetrance between 0.41 and 1.11% [36].  Table 4 also shows that 10 of the 26 cancer cases (38.5%) were below 45 years old which is far lower than the 69.5% we previously reported [18]. The previous study included five regions in Ghana of which central region was not included. Moreover, the region in the previous study that had an extreme high prevalence was not included in this study. It is therefore important that we extend the study with Breast-i to all regions in Ghana and a proposal has been drawn for the Ghanaian government.

## 5. Conclusion

In conclusion, the study set out to answer questions such as whether Breast-i will be an effective alternative screening tool especially in dense, dark breasts of younger Black women, whether it can pick up suspicious lesions missed by CBE, and whether it can be recommend for usage in the population as an adjunct screening device. Our results reveal that Breast-i is a much more effective screening device especially by being able to pick up both nonpalpable lesions and very small lesions in larger breast which are often missed by CBE. Due to detection of angiogenesis, Breast-i pickups always warrant further evaluation as it is potentially cancerous. Furthermore, being battery operated and emitting no radiation, Breast-i can be used on all women from 15 years and above, pregnant or lactating. Finally, due to its high sensitivity and specificity coupled with the screening procedures being more acceptable by women, we will recommend Breast-i for both routine and mass screening in Ghana and possible other sub-Saharan countries. We see Breast-i as the future of screening and early detection of breast cancer in sub-Saharan countries to reduce late presentations and improve survival.

## Figures and Tables

**Table 1:** 

	Central region	Greater Accra region
Total participants	1460	744

Average age (mean)	34	41

*Education level*		
Primary/none	262	136
Secondary	430	396
Tertiary	768	212

Urban dwelling	1067	682
Rural dwelling	393	62

*Knowledge level of breast cancer*		
None	22	0
Little	365	322
Above adequate	1073	422

**Table 2:** Suspicious cases as per procedure of detection.

	Total participants	Suspicious lumps palpated and detected by Breast-i	Suspicious lesions detected by Breast-i only	Total detected by each procedure	No Abnormalities detected
Breast-i	2204	136	22	158	2046
CBE	2204	153	-	153	2051

**Table 3:** Suspicious cases that were confirmed as cancerous.

	Total suspicious masses	Nonpalpable but angiogenic	Small lumps in large breast	Detection by Breast-i and CBE	Total cases detected per procedure	Total confirmed cases
Breast-i	158	4/7	3/15	17	24	26
CBE	153	-	-	19	19	26

**Table 4:** Breast cancer cases in relation to numbers and age.

	<25	25–34	35–44	45–54	55–64	>64	Total
Number of participants	346	728	490	418	170	52	2204
Breast cancer cases	1	3	6	10	4	2	26

## Data Availability

The datasets used and/or analyzed during the current study are available from the corresponding author upon reasonable request.
